# Mesenchymal Stem Cells Repress Th17 Molecular Program through the PD-1 Pathway

**DOI:** 10.1371/journal.pone.0045272

**Published:** 2012-09-17

**Authors:** Patricia Luz-Crawford, Danièle Noël, Ximena Fernandez, Maroun Khoury, Fernando Figueroa, Flavio Carrión, Christian Jorgensen, Farida Djouad

**Affiliations:** 1 Inserm, U 844, Montpellier, France; 2 Université Montpellier 1, UFR de Médecine, Montpellier, France; 3 Service d’Immuno-Rhumatologie, Hôpital Lapeyronie, Montpellier, France; 4 Laboratorio de Immunologia Celular y Molecular, Universidad de Los Andes, San Carlos de Apoquindo, Santiago, Chile; 5 Programa Doctorado en Biotecnologia, Universidad Santiago de Chile, Avenida Libertador Bernardo O’Higgins, Santiago, Chile; New York University, United States of America

## Abstract

MSC display potent suppressive properties initially described a decade ago. More recently, MSC suppressive activities on T-cell effector pathways have been investigated. MSC modulate CD4 differentiation through different mechanisms depending on culture conditions and display disparate activities on T cells according to their differentiation status. A significant amount of evidence for MSC effects on Th17 cells revealed that MSC could be suppressive under diverse circumstances but also enhance Th17 cell activity under other conditions. In the present study, we investigated the suppressive effects of MSC on Th1 and Th17 subsets of T cells using T cells undergoing Th1 and Th17 polarization or mature Th1 and Th17 cells. MSC inhibited the proliferation of T cells during their differentiation toward Th1 cells and mature Th1 cells. This suppressive effect was maintained in a transwell cell culture insert demonstrating the major role played by soluble factors. Using the transwell cell separation barrier, we observed that MSC decrease the number of T cells undergoing Th17 differentiation whereas they did not affect IL-17 production by mature Th17, demonstrating the need for cell contact for suppressing Th17 cell function. Moreover, we reported that PD-L1 is highly expressed on MSC co-cultured with differentiating or polarized Th1 and Th17 cells. Using neutralizing antibodies specific for PD-L1 and PD-1 we showed that the mechanisms by which MSC mediate Th17 cell repolarization depend on PD-L1 expression on MSC. Taken together our results demonstrated a cell-to-cell contact depend mechanism in the selective immunosuppression of MSC on mature Th17 cells through up-regulation of PD-L1.

## Introduction

Multipotent mesenchymal stromal cells or mesenchymal stem cells (MSC) are progenitor cells essentially isolated from bone marrow or adipose tissue [1]. Besides their capacity to differentiate into various cell lineages such as chondrocytes, osteoblasts or adipocytes, MSC display potent T-cell suppressive properties initially described a decade ago both *in vitro* and *in vivo* [2]–[4]. The mechanisms by which MSC exert their immunosuppressive effect are beginning to be elucidated underlying substantial differences in immune biology between mouse and human MSC. Both cell-to-cell contact and secretion of soluble factors have been proposed. The possible mediators identified include factors idiosyncratic to mouse MSC such as inducible nitric oxide synthase (iNOS) [5], to human MSC including indoleamine 2,3-dioxygenase (IDO) [6] and human leukocyte antigen (HLA)-G [7] and overlapping factors without discrimination such as prostaglandin E2 (PGE2) [8], [9]. MSC suppressive properties are mediated through the action of these latter factors and result in the inhibition of the proliferation of CD4^+^ and CD8^+^ T cells, B lymphocytes, NK cells that has been mainly described *in vitro* but also *in vivo* in a number of experimental models [8], [10]–[13].

T and B cell activation was shown to be suppressed by cell-to-cell contact, while soluble factors were effective in inhibiting B lymphocyte proliferation [10]. However, the precise mechanism of action of MSC-mediated immunosuppression remains controversial, in part, due to the use of mixed populations of splenocytes or lymphocytes in the studies. Few reports have addressed the effect of MSC on specific T cell subsets. To date, it has been described that MSC inhibit the differentiation toward the Th1 lineage *in vitro* and *in vivo* and induce the generation of regulatory T cells [14]–[16]. Effects of MSC on the pro-inflammatory Th17 cells are more controversial. In various experimental models of Th17-derived autoimmune diseases, administration of MSC has been shown to suppress inflammation and autoimmunity [17]–[19]. *In vitro*, we previously showed that MSC induce Th17 cells to exhibit regulatory T cell characteristics in an inflammatory environment [20]. Although these studies suggest that MSC suppress Th17 cell activity, some evidence for a Th17 cell-activating effect of MSC has also been observed. For example, it has been shown that MSC-conditioned media suppresses Th1 cell activity while increasing Th17 responses [21]. Moreover, MSC produce IL-6 and TGF-β1 when co-cultured with activated T cells, suggesting that they may induce Th17 cell differentiation. All together these studies reveal that depending on environmental conditions, MSC can exert either suppressive or enhancing effect on Th17 cell activity.

The role of soluble factors in MSC-mediated suppression has been largely investigated. For example, MSC suppress Th1 differentiation and function mainly through indirect mechanisms [15], [22]. The dual role of PGE2 on T cell differentiation and proliferation was described on different T helper cell subsets. Indeed, according the context and the target of its action, PGE2, can display either pro-inflammatory or anti-inflammatory properties. PGE2 exerts an inhibitory effect on mature Th1 cell proliferation and production of IFN-γ, on one hand, and enhances IFN-γ production during the differentiation of naïve T cells toward Th1 lineage, on the other hand [23]–[26]. In contrast, while PGE2 has been shown to enhance IL-17 production by polarized Th17 cells it suppresses IL-17 during Th17 cell differentiation [27]–[29]. Therefore, controversial data obtained regarding the immune modulation capacity of MSC could also be explained by the dual role of soluble factors released by MSC on the function of distinct T cell subsets.

The role of cell-to-cell contact has also been proposed to be necessary for MSC immunosuppressive effect. Programmed death-1 (PD-1) is a molecule expressed on various cell types, including a subset of thymocytes and activated T and B cells that plays an important role in the negative regulation of immune responses and the maintenance of peripheral tolerance http://www.nature.com.gate2.inist.fr/gt/journal/vaop/ncurrent/full/gt2011185a.html - bib5[30]. PD-1 ligand, PD-L1, broadly expressed in different tissues, has been shown to be further induced by exposure to interferon (IFN)-γ on several tumour cell lines and MSC [11], [31]–[33]. The subsequent primed MSC, in turn, suppress T cell proliferation via PD-L1-mediated inhibitory signaling [10], [11]. Therefore, IFN-γ could play a critical role in MSC-mediated immunosuppression through up-regulation of PD-L1 providing evidence of a cell contact-dependent mechanism. However, whereas English and collaborators confirmed that IFN-γ induces PD-L1 protein expression by MSC, they have shown that MSC mediated suppression did not involve an essential role for PD-1 pathway [34]. The reasons for these differences are most likely due to the use of different stem cell populations and immune cells. Indeed, the authors used not only MSC with different phenotypes but also heterogeneous population of immune cells such as splenocytes or lymphocytes. All together these studies suggest that depending on the targeted T cell subsets, different molecular mechanisms are involved in MSC-mediated immunosuppressive action.

Herein we assessed for the first time, in the same report, MSC suppressive effect on different polarized T-helper-cell subsets. Indeed, we designed the study to elucidate the effect of MSC 1) during the differentiation of CD4^+^ T cells toward Th1 and Th17 lineages or, 2) on mature Th1 and Th17 cell subsets. Moreover, we investigated the role of soluble factors or cell-contact on the suppressive effects of MSC. In this study, we demonstrate that MSC suppressive effect on mature Th17 cell function and proliferation is contact dependent and mediated by PD-L1 up-regulation on primed MSC.

## Materials and Methods

### Ethics Statement

C57BL/6 and Female DBA/1 mice were obtained from the breeding unit of our animal facility (French Health Authorities agreement n°B34-172-36). The experimental protocol including these mice was approved (CEEA-LR-1067 and CEEA-LR-10042) by the Languedoc-Roussillon Ethical Committee for Animal Experimentation (registered as n°36 of the French National Committee for Ethical Consideration on Animal Experimentation).

The animals were maintained in accordance with national guidelines for animal care. Bedding was enriched with wood shaving for nesting and cages were provided with igloos for breeding. Animals used in this study did not receive any treatment. They were euthanized, at the age of 10 weeks, by isoflurane inhalation and cervical decapitation, before collecting organs (femurs, tibias and spleens).

### Isolation of Mesenchymal Stromal Cells

MSC were isolated and fully characterized from C57BL/6 mice as described previously [18] following the mesenchymal stem cell minimal criteria [35]. Briefly, bone marrow was flushed out of long bones and the cell suspension was plated (0.5×10^6^ cells/cm^2^) in minimum essential medium (MEM)-α supplemented with 10% fetal bovine serum (FBS) (Hyclone, Thermo Fisher Scientific, Brebières, France), 2 mM glutamine, 100 U/mL penicillin, 100 mg/mL streptomycin (Lonza, Levallois-Perret, France) and 2 ng/mL human basic fibroblast growth factor (bFGF) (R&D Systems, Lille, France). At sub-confluence, cells were replated at the density of 5,000 cells/cm^2^ and used between passage 7 and 12 [18]. The capacity of MSC to differentiate toward the chondrogenic, adipogenic and osteogenic lineages was tested.

### Differentiation Induction

CD4^+^ T cells were isolated from spleen of DBA/1 mice by negative selection using Dynal® CD4 negative isolation kit (Invitrogen, Saint Aubin, France) according to the manufacturer’s instructions. Purified CD4^+^ T cells were cultured in complete IMDM medium containing 10% of heat-inactivated FBS, 2 mM L-glutamine, 100 U/mL penicillin, 100 µg/mL streptomycin, 0.1 mM non essential amino acids, 1 mM sodium pyruvate, 20 mM HEPES and 50 µM of beta-mercaptoethanol (Invitrogen, Saint Aubin, France).

T cells were stimulated with anti-mouse CD3/CD28 Dynabeads (Invitrogen, Saint Aubin, France) and cultured alone or, in contact with MSC or, physically separated from MSC using a 0.4 µm porous transwell system (Corning Incorporated, Life Sciences, France). MSC were added at the ratios 1∶10 or 1∶100, at day 0 or day 4 of the T cell differentiation processes. Th1 differentiation was induced by adding 20 ng/mL IL-12 and 2.5 µg/ml anti–IL-4 antibody while Th17 priming was induced using 2.5 ng/mL TGF-β1, 50 ng/mL IL-6, 2.5 µg/mL of both anti-IFNγ, and anti–IL-4 antibodies (R&D Systems, Lille, France). After 6 days of culture, cell proliferation was measured using the CellTiter-Glo™ luminescent cell viability assay (Promega, Charbonnières-les-Bains, France) and intracellular cytokine staining was performed.

When indicated, neutralizing antibodies against PDL-1 or PD-1 (eBiosciences, Paris, France) were added to the co-cultures at day 0 or 4 of the T cell differentiation processes at a concentration of 10 µg/mL.

### RT-qPCR Analysis

Total RNA was extracted using the RNeasy mini kit (Qiagen S.A., Courtaboeuf, France). RNA was reverse transcribed using the Multiscribe reverse transcriptase (Applied Biosystems, Courtaboeuf, France). PCR was performed using the SYBR Green I Master kit according to the manufacturer’s recommendations (Roche Applied Science, Meylan, France). Analysis of mRNA expression level was performed using the Roche LightCycler^®^ 480 software1.5. Expression level of transcripts was normalized to endogenous ribosomal protein S9 (RPS9) mRNA levels (reference gene) according to the formulae 2^−ΔCt^.

### Antibodies and FACS Analysis

The following monoclonal antibodies (mAbs) were purchased from BD Pharmingen (Le Pont-de-Claix, France): PE-conjugated anti-IFN-γ mAb and isotypic control, PerCP-conjugated anti-IL-17 mAb and isotypic control. APC-conjugated RORγT, PE-conjugated anti-PDL1 and anti-PD1 mAbs were purchased from eBiosciences (Paris, France). After membrane and intracellular staining, cells were analyzed on FACS CantoII using the BD FACSDiva software.

### Measurement of iNOS Activity

Because nitric oxide (NO) is quickly converted to NO_2_ and NO_3_ in culture medium, we measured NO_2_ production using a modified Griess reagent (Sigma-Aldrich) as previously described [36].

### Quantification of Cytokines

Cytokines were quantified in culture supernatants from T-cell proliferation assays. Supernatants were collected and stored at −20°C until tested for the presence of cytokines. IL-10 was quantified using an ELISA from R&D systems (Lille, France) and PGE2 using the PGE2 ELISA from Arbor Assays (Souffelweyersheim, France).

### Statistical Analysis

Individual experiments were carried out between 3 and 7 times to ensure reproducibility. Results are expressed as the mean ± standard error of the mean (SEM). *P* values were generated by ANOVA. Multiple comparisons were corrected by Bonferroni test or the Dunnett test (***P<0.001, **P <0.01 and, *P <0.05).

## Results

### Inhibition of Th17 Cell Proliferation and Function by MSC is Dose-dependent

**Figure 1:**
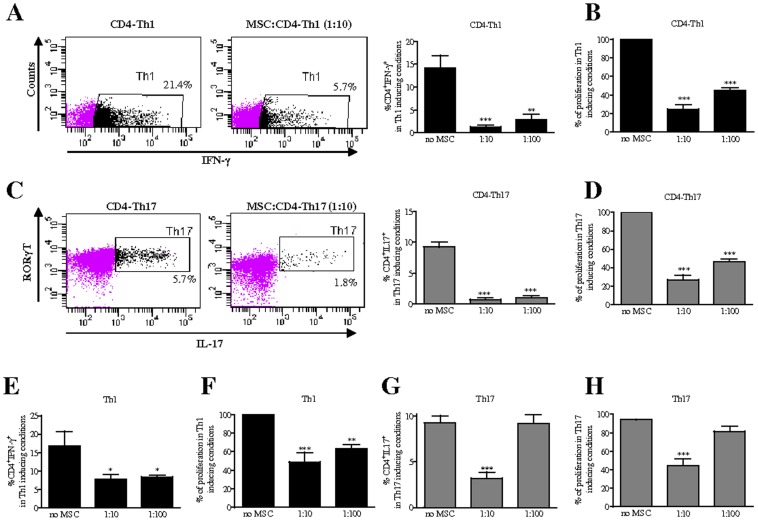
Dose-dependent inhibition of mature Th17 cells by MSC. (A, C, E and G) Cell differentiation and (B, D, F and H) proliferation were assessed using T cells induced to differentiate into Th1 or Th17 cells in the absence or presence of MSC added at day 0 (CD4-Th1 or CD4-Th17, respectively) (A, B, C and D) or at day 4 of the differentiation process (Th1 or Th17, respectively), at two MSC:T ratios 1∶10 and 1∶100. (E, F, G and H). Results are expressed as the percentage of anti-CD3/CD28-induced proliferation of T cells which was assigned the value of 100%. We used the Cell Titer GloTM luminescence assay to quantify T cell proliferation and flow cytometry intracellular detection of IFN-γ (Th1) or RORγT and IL17 (Th17) for differentiation (A and C, representative dot plots). *P* values referred to the condition without MSC (no MSC).

First, the effect of MSC on the polarization and proliferation of naïve CD4^+^ T cells toward the Th1 and Th17 lineages (CD4-Th1 or CD4-Th17) was investigated using purified CD4^+^ T cells induced to differentiate *in vitro* following stimulation by anti-CD3/CD28 beads in the presence of IL-12 and anti–IL-4 for Th1 priming and TGF-β1, IL-6, anti-IFNγ, and anti–IL-4 for Th17 priming. Consistent with reports in the literature, these combinations of cytokines and antibodies induced, respectively, the generation of a population of IFN-γ-producing cells and IL-17-producing cells positive for the Th17 lineage-specific transcription factor RORγT (**Fig. 1A and 1C**). The addition of MSC at day 0 of the differentiation process resulted in the inhibition of T cell proliferation which was associated with a significant decrease of IFN-γ-producing Th1 cells (**Fig. 1A and 1B**). This effect was observed at the two MSC:T cell ratios tested. A similar inhibitory effect of MSC on T cell induced to differentiate toward the Th17 lineage was obtained (**Fig. 1C and 1D**). We then assessed the effect of MSC on mature Th1 or Th17 cells. The suppressive effect of MSC on the number of mature Th1 cells and their proliferation was effective at MSC:T cell ratios of 1∶10 and 1∶100 (**Fig. 1E and 1F**). However, while this suppression mediated by MSC was observed on mature Th17 cells at the MSC:T cell ratio of 1∶10, mature Th17 cell proliferation as well as their IL-17 production capacity were not affected at the ratio 1∶100 (**Fig. 1G and 1H**). All together, these results suggested that MSC exert a stronger immunosuppressive effect on the Th1 lineage compared to the Th17 cell subset.

### Expression of TGF-β1, NO_2_ and PGE2 by MSC when Cultured under Th1- and Th-17 Skewing Conditions

**Figure 2:**
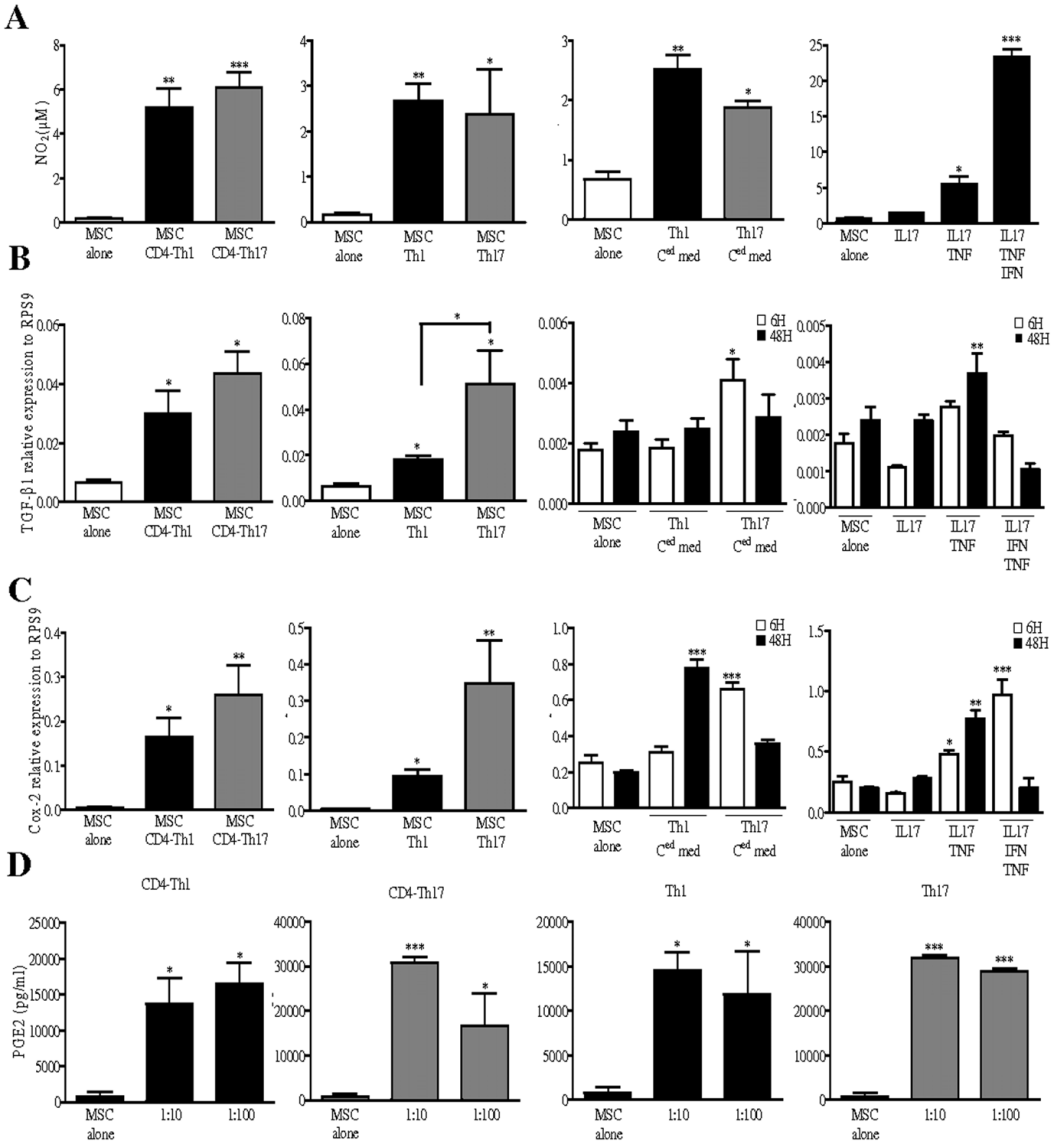
Th1 and Th17 cells up-regulate NO, TGF-β1 and Cox2 expression levels in MSC. (A) Quantification of NO_2_ using a modified Griess reagent and (D) PGE2 secretion by ELISA. Levels of NO_2_ and PGE2 were quantified in the supernatants of MSC co-cultures with either T cells induced to differentiate toward Th1 or Th17 lineages (CD4-Th1 and CD4-Th17) or with mature Th1 and Th17. (A) Quantification of NO_2_ in the supernatant of MSC treated for 48 hours with Th1- or Th17-conditioned media. T-cell-conditioned medium (C^ed^ med) were obtained from a 3-day culture of either differentiated Th1 or Th17 cells. Quantification of NO_2_ in the supernatant of MSC treated during 48 hours with IL-17 alone or in combination with either TNF-α or TNF-α and IFN-γ. (D) PGE2 was quantified in the supernatant of T cells co-cultured with MSC (MSC:T cell ratios 1∶10 and 1∶100). (B) Evaluation of TGF-β1 and (C) Cox-2 expression levels by quantitative real time PCR. (B and C) mRNA was extracted from MSC after co-culture with CD4^+^ T cells undergoing Th1 or Th17 lineages or with mature Th1 or Th17 cells. (B) TGF-β1 and (C) Cox-2 expression levels were quantified in MSC after a 6 or 48 hours treatment with Th1- or Th17-conditioned media. (B) TGF-β1 and (C) Cox-2 expression levels in MSC were also assessed after a 6 or 48 hours treatment with IL-17 alone or in combination with either TNF-α or TNF-α and IFN-γ. If not indicated, *P* values referred to the condition when MSC are cultured alone (MSC alone).

We then investigated whether the molecules described in the literature to be involved in MSC immunosuppressive effects could explain the discrepancies observed here between Th1 and Th17 mature cells. Herein, we showed that T cells undergoing Th1 or Th17 differentiation as well as mature Th1 and Th17 cells can induce the secretion of NO_2_ by MSC (**Fig. 2A**). Secretion of NO_2_ by MSC was also observed when cells were cultured for 48 hours with either Th1- and Th17-conditioned media. In both cases, MSC secreted similar amount of NO_2._ To define which cytokines produced by the differentiated T cells were responsible for NO_2_ secretion, we treated MSC during 48 hours with IL-17 *±* TNF-α *±* IFN-γ. IL-17 alone did not affect NO_2_ secretion whereas in combination with TNF-α, it led to a slight up-regulation of NO_2_ production. Moreover, IFN-γ acted in synergy with IL-17 and TNF-α combination to significantly increase NO_2_ secretion (**Fig. 2A**). Then, we looked at TGF-β1 and COX-2 mRNA expression level in MSC and found out that both activated CD4^+^ T cells under Th1- or Th17-skewing conditions and mature Th1 and Th17 cells significantly increased TGF-β1 and COX-2 mRNA (**Fig. 2B and 2C**). Interestingly, Th17 cells induced higher levels of TGF-β1 in MSC than Th1 cells. Increased expression levels of TGF-β1 mRNA in MSC were also observed when MSC were primed with Th17 conditioned media. However, Th1 conditioned media did not affect TGF-β1 expression level in MSC suggesting that TGF-β1 expression could not explain the more potent inhibitory effect of MSC on Th1 cells (**Fig. 2B**). On the contrary, COX-2 mRNA levels were increased in MSC when co-cultured with either Th1 or Th17 conditioned media suggesting that soluble factors released by these two cell types stimulate COX-2 expression (**Fig. 2C**). No significant difference in COX-2 expression level in MSC was observed between Th1 or Th17 stimulating culture conditions. To address the role of Th1 and Th17 cell-specific cytokines in the up-regulation of TGF-β1 and COX-2, we used the corresponding recombinant cytokines in similar conditions. As shown, IL-17 addition did not affect the expression level of these factors in MSC. However, after 48 hours of treatment with both IL-17 and TNF-α; TGF-β1 and COX-2 were up-regulated in MSC (**Fig. 2B and 2C**). Intriguingly, while the addition of IFN-γ to the latter combination significantly reduced TGF-β1 expression level by MSC, it induced a higher expression of COX-2 (**Fig. 2B and 2C**). Since COX-2 is an inducible enzyme involved in the production of PGE2, we then focused our attention on PGE2 secretion by MSC when primed with differentiating or mature Th1 or Th17 cells. As shown, compared with MSC alone, MSC secreted higher levels of PGE2 when co-cultured with T cells induced to differentiate toward the Th1 or Th17 lineages or with mature Th1 or Th17 cells, whatever was the ratio tested, though to a lesser extend for differentiating and mature Th1 cells (**Fig. 2D**). These results revealed that neither TGF-β1 nor NO_2_ or PGE2 secretion can explain why MSC display unequal inhibitory effect on Th1 and Th17 cells.

### The Suppressive Effect of MSC on Th17 Cell Molecular Identity is Contact-dependent

**Figure 3:**
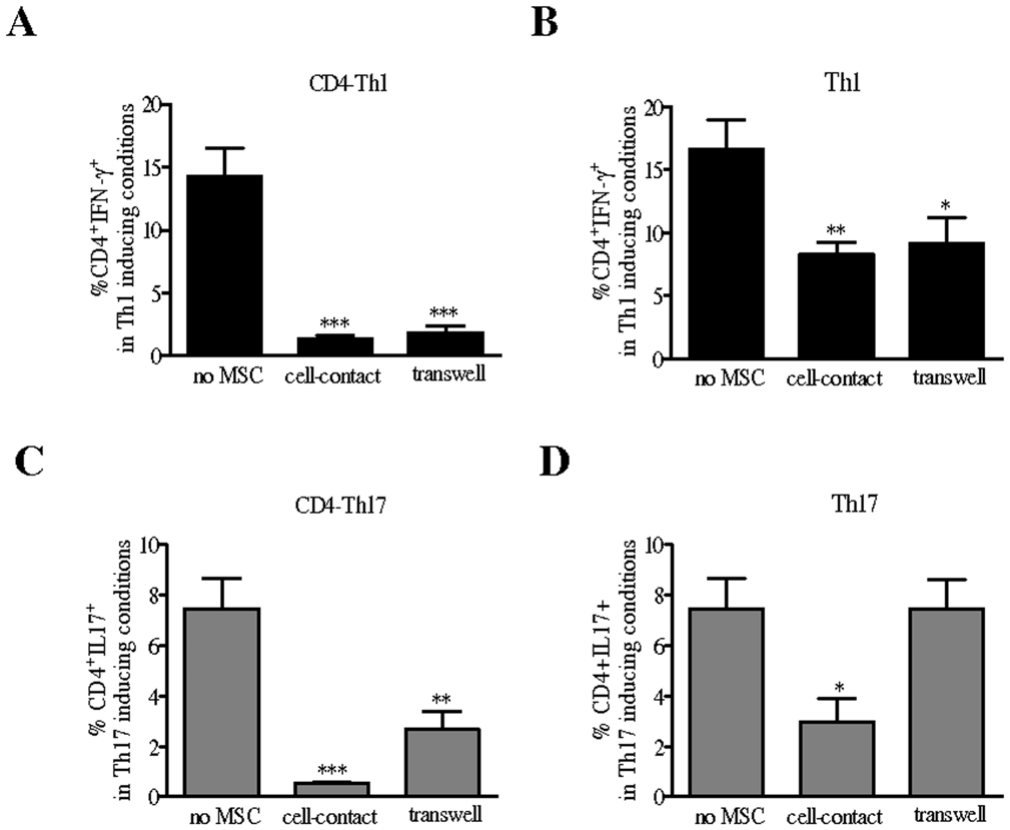
Contact-dependent inhibition of mature Th17 cells by MSC. Effect of MSC on CD4^+^ T cell differentiation toward (A) the Th1 or (C) Th17 lineages (CD4-Th1 or CD4-Th17, respectively) or on mature (B) Th1 or (D) Th17 cells when cultured in absence of a physical contact between the cells using a transwell system (MSC:T cell ratio of 1∶10). *P* values referred to the condition without MSC (no MSC).

We then investigated whether the differential effect mediated by MSC on Th1 and Th17 cells was due to cellular contact. The requirement for cell-cell contact was examined at the MSC:T-cell ratio of 1∶10 using transwell chambers in which T cells undergoing Th1 or Th17 induction or mature Th1 or Th17 cells were cultured in the lower compartment and separated from MSC in the upper compartment. As shown in figure 3, the suppressive effects of MSC on the number of IFN-γ-producing CD4^+^ T cells during Th1 differentiation or mature Th1 cells, were fully maintained in a transwell cell-culture system (**Fig. 3A and 3B**). However, using the same culture conditions, we found out that while MSC significantly decreased the percentage of IL-17-secreting CD4^+^ T cells undergoing Th17 induction, they did not affect the number of IL-17 producing mature Th17 cells (**Fig. 3C and 3D**). All together these results indicated that cell contact is required for MSC to repress the Th17 molecular program.

### T Cells Undergoing Th1 and Th17 Differentiation as well as Mature Th1 and Th17 Cells Up-regulate PD-L1 Expression on MSC

**Figure 4:**
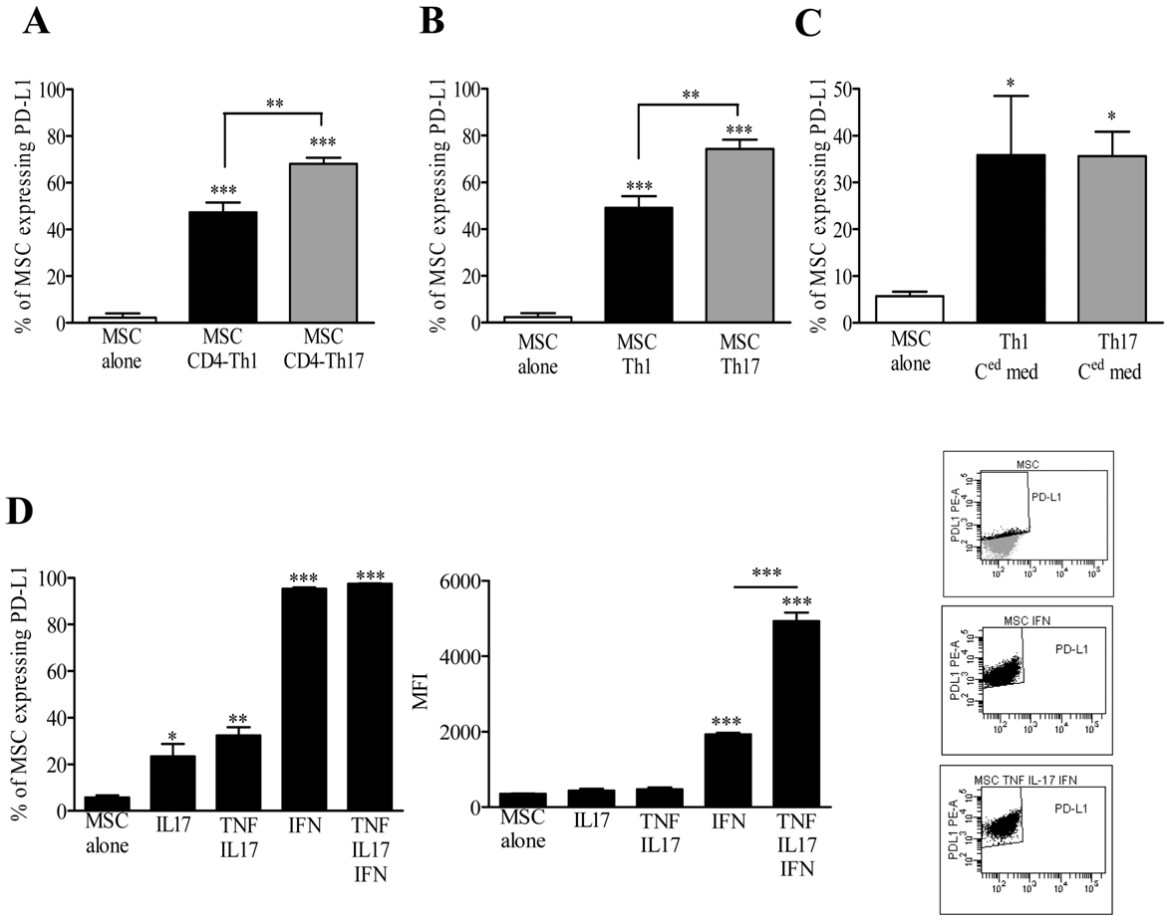
Th1 and Th17 cells induce PD-L1 expression on MSC. (A) Percentage of MSC expressing PD-L1 after co-culture with activated T cells undergoing Th1 or Th17 lineages (CD4-Th1 or CD4-Th17, respectively) or (B) with mature activated Th1 or Th17 cells, detected by flow cytometry. (C) PD-L1 molecule expression profile on MSC after 6 hours of incubation with Th1- or Th17-conditioned media. (D) The percentages of MSC positive for PD-L1 and the mean fluorescence intensities (MFI) upon a 6 hours treatment with IL-17 and IFN-γ alone or in combination with TNF-α and IFN-γ. On the right, representative flow cytometry dot plots illustrating PD-L1 protein expression on untreated control and treated MSC. If not indicated, *P* values referred to the condition corresponding to MSC alone.

Controversy studies have shown that PD-L1 up-regulation by IFN-γ could play or not a role in the immunosuppressive properties of MSC via direct contact with activated mouse splenocytes or lymphocytes [10], [11], [34]. To go further, we investigated the role of Th1 and Th17 cell culture conditions in priming MSC through up-regulation of PD-L1. Flow cytometry analysis revealed that PD-L1 was significantly up-regulated on MSC after co-culture with T cells undergoing Th1 or Th17 differentiation (**Fig. 4A**) or with mature Th1 or Th17 cells (**Fig. 4B**). However, under Th17 culture conditions we observed a significantly higher increased of PD-L1 compared to Th1 conditions. Using the conditioned media obtained from the cultures of mature Th1 and Th17 cells we also observed a significant increase of PD-L1 expression on MSC (**Fig. 4C**). We then investigated which cytokine was responsible for PD-L1 up-regulation on MSC, and showed that IL-17 alone or in combination with TNF-α, increased the percentage of MSC expressing PD-L1. Moreover, addition of IFN-γ alone or in combination with TNF-α and IL-17 induced a significantly higher percentage of PD-L1 positive MSC. While the percentage of positive cells reached almost 100% in both cases, we showed that the mean fluorescence intensity (MFI) was significantly higher in MSC treated with IFN-γ in combination with TNF-α and IL-17 compared to IFN-γ alone (**Fig. 4D**).

### PD-1 Expression Increases on T Cells during Th1 and Th17 Differentiation Independently of MSC

**Figure 5:**
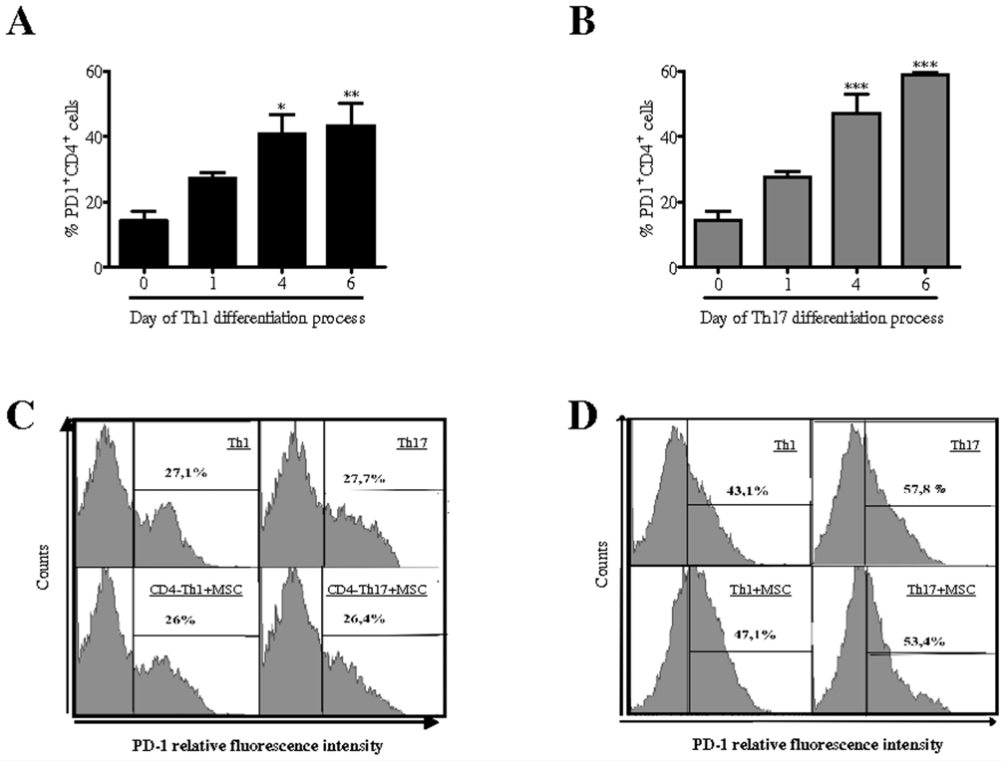
PD-1 expression profile on T cells undergoing Th1 or Th17 differentiation is not modified by the co-culture with MSC. Percentage of PD-1 expressing T cells during their differentiation toward (A) Th1 or (B) Th17 lineages (day 0, 1, 4 and 6 of the differentiation process) was detected by flow cytometry. Percentage of PD-1 expressing T cells in the presence or absence of MSC added either (C) at day 0 of the differentiation process or (D) on mature Th1 and Th17 cells. *P* values referred to PD-1 expression level on purified CD4^+^ T cells at day 0 of differentiation processes.

Induction and maintenance of T cell tolerance require PD-1 expression on T cells and its activation by PD-L1 expressed on nonhematopoietic cells to limit effector T cell responses [37]. Since PD-1 is up-regulated on T cells upon activation and that PD-L1 can inhibit T cell responses by promoting the induction of regulatory T cells [38], we investigated whether MSC could influence PD-1 expression on T cells undergoing Th1 and Th17 differentiation and on fully differentiated Th1 and Th17 cells. First, we showed that PD-1 expression level was increased gradually during the Th1 (**Fig. 5A**) and Th17 differentiation (**Fig. 5B**) processes to reach the maximum at day 4. Interestingly, we found out that this up-regulation of PD-1 expression level on CD4^+^ T cell induced to differentiate toward the Th1 and Th17 lineages (**Fig. 5C**) or on mature Th1 and Th17 cells (**Fig. 5D**) was not affected by the presence of MSC. Thus, MSC did not exert their suppressive properties by modulating PD-1 expression on T cells.

### PD-1 Pathway Plays a Critical Role in MSC-mediated Repression of the Th17 Molecular Program

**Figure 6:**
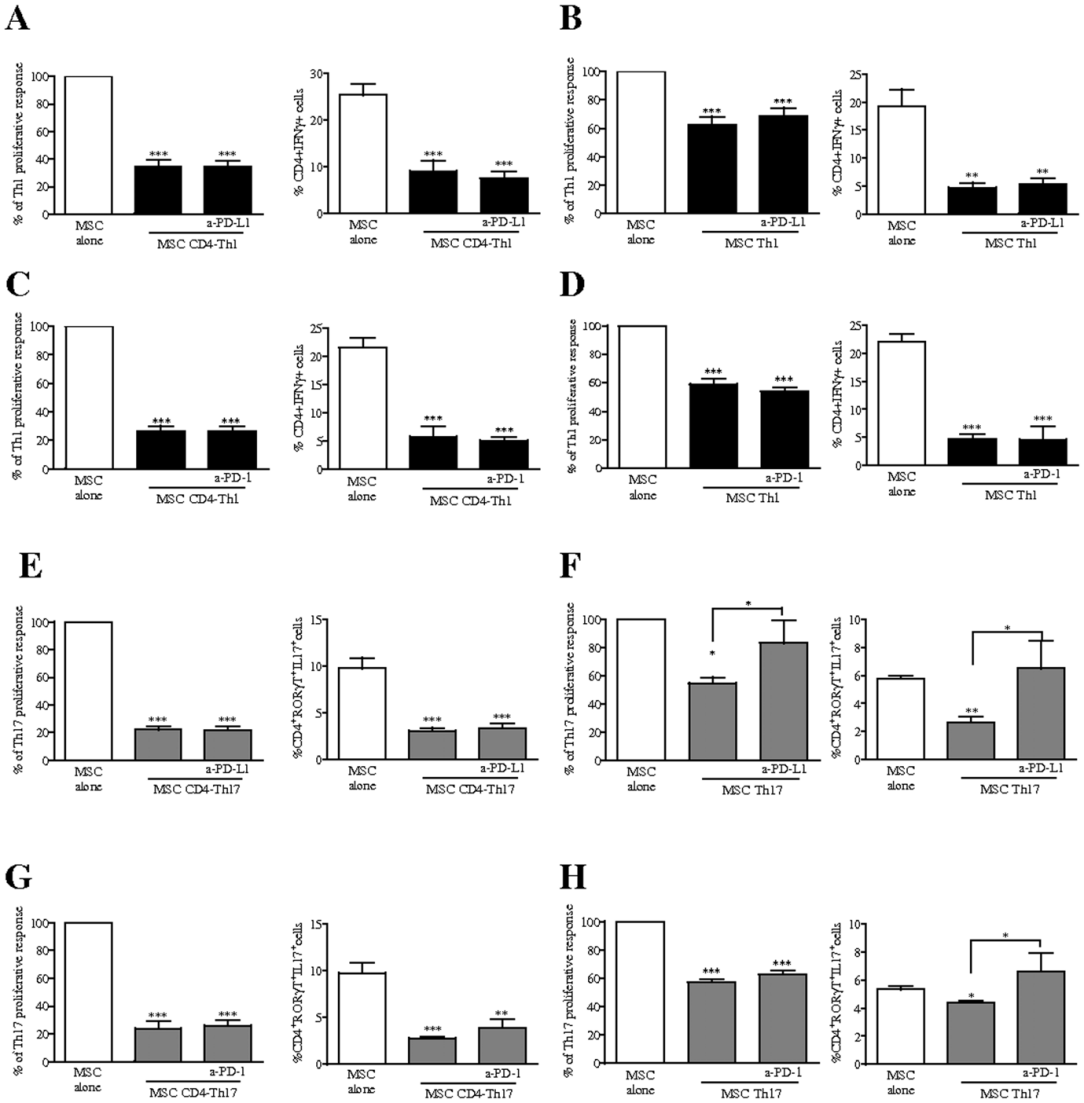
PD-L1 blockade partially reverse the immunosuppressive properties of MSC on mature Th17 cells. We assessed the effect of MSC on the proliferation and differentiation of activated CD4+ T cells induced to differentiate into (A and C) Th1 (CD4-Th1) and (E and G) Th17 (CD4-Th17) cells and on mature (B and D) Th1 and (F and H) Th17 cells in the presence or absence of 10 µg/ml of neutralizing antibodies against PD-L1 or PD-1. We used the Cell Titer GloTM luminescence assay to quantify T cell proliferation and flow cytometry intracellular detection of IFN-γ (Th1) or RORγT and IL17 (Th17) for differentiation. Proliferation results are expressed as the percentage of anti-CD3/CD28-induced proliferation of the different T cell subsets which was assigned the value of 100%. If not indicated, *P* values referred to values obtained for MSC when cultured alone (white bars). CD4^+^ T cells undergoing Th1 lineage and mature Th1 cells are represented with black bars. CD4^+^ T cells undergoing Th17 lineage and mature Th17 cells are represented with grey bars.

To obtain direct evidence that PD-L1 induction on MSC is critical for their immunomodulatory function, we used neutralizing PD-L1 mAb. As shown in figure 6A, in co-cultures of MSC with activated T cells induced to differentiate toward the Th1 lineage, PD-L1 blockade did not affect the percentage and proliferation of Th1 cells (**Fig. 6A**). Consistent with these results, we found out that PD-L1 blockade did not affect the capacity of MSC to decrease the frequency of IFN-γ producing T-cells when co-cultured with differentiating and mature Th1 cells (**Fig. 6A and 6B**). Similar results were obtained using a neutralizing PD-1 mAb on T cells (**Fig. 6C and 6D**). Interestingly, in the presence of the neutralizing antibody to PD-L1, while MSC inhibited the proliferation of activated T cells undergoing Th17 differentiation (**Fig. 6E**), they failed to suppress the proliferation of activated fully differentiated Th17 cells (**Fig. 6F**). In parallel, we demonstrated that PD-L1 expression by MSC is not required for inhibiting the generation Th17 cells producing IL-17 and positive for RORγT (**Fig. 6E**) but is necessary to mediate their repressive function on mature Th17-gene program (**Fig. 6F**). We also performed blocking experiments using the antibody specific for PD-1 and obtained equivalent effects (**Fig. 6G and 6H**). All together, these results revealed that PD-1/PD-L1 pathway is critical for MSC-mediated repression of mature Th17-gene program and cell function in favour of alternative T helper cell subtypes.

## Discussion

The immunosuppressive function of MSC is mediated by multiple pathways and blocking only one pathway is unlikely to inhibit the complete suppressive properties of MSC. Both cell-to-cell contact and soluble factors have been demonstrated to be implicated in MSC-mediated immunomodulatory properties. Taking into account the fact that mouse and human MSC may use different pathways to regulate activity of immune cells, we decided, in the present study, to focus our attention on mouse MSC. We investigated the capacity of MSC to interfere with the functional activity of two subsets of CD4^+^ T cells; IFNγ- and IL17-producing Th1 and Th17 cells, respectively, which play an important role in the pathogenesis of several autoimmune and inflammatory diseases. Using activated CD4^+^ T cells cultured under *in vitro* conditions for Th1 and Th17 lineage differentiations, we showed that MSC inhibited the proliferation of the activated cells as well as the cytokine production specific for Th1 and Th17. The same immunomodulatory action of MSC on the proliferation and the cytokine production profile was observed on fully differentiated Th1 and Th17 lymphocytes. Importantly, the present report reveals that MSC mediate a phenotypic switch from mature Th17 cells to alternative T helper subset characterized by the repression of Th17 molecular program through a cell contact-dependent mechanism.

In our previous study using human MSC, we showed that the effect of MSC on the inhibition of IL-17 production by mature Th17 cells was abolished in a transwell cell-culture system indicating that cell contact is required for their inhibitory activity [20]. In the present study, using transwell cell-culture inserts, we showed that cell contact is not necessary for murine MSC to suppress the differentiation of activated CD4^+^ T cells into Th1 or Th17 cells or to convert the phenotype of fully differentiated Th1 cells. However, we confirmed that a cell-to-cell contact is required for MSC-mediated repression of mature Th17-gene program in favor of an alternative noninflammatory T helper cell subtype. Therefore, MSC exert their immunomodulatory properties by means of different mechanisms. Indeed, while MSC exercise their suppressive effects on Th1 cells through the secretion of soluble factor(s), the mechanism mediating the inhibitory potential of MSC on Th17 is cell-contact dependent. It is now well established that PGE2 produced by MSC in direct contact with T cells partly mediates their potent suppressive effect [39], [40]. However, PGE2 can have disparate activities on T cells depending on their differentiation status [27], [29]. In the present study, similar amounts of PGE2 were produced in the co-cultures of MSC with Th1 or Th17 cells, both during their differentiation or when they were fully mature. We also showed that MSC cultured with T cells induced to differentiate into Th1 and Th17 cells and to a lower extend with fully differentiated Th1 and Th17 cells produced increased levels of NO_2_. However, we did not observe any significant difference between Th1- or Th17-skewing conditions. Therefore, neither PGE2 nor NO_2_ secretion can explain the differential immunoregulatory potential of MSC on Th1 and Th17 cell proliferation and phenotype.

Augello et al. found out that MSC cultured on transwell membranes with PHA-stimulated splenocytes significantly increased the mRNA expression level of TGF-β1 and TGF-β1 receptor [10]. Moreover, the suppressive effect of MSC on primary Th17 induction was suggested to be mediated by the up-regulation of COX-2 in MSC following direct contact with activated T cells [40]. This is consistent with our previous report showing that COX-2 inhibition by indomethacin reversed MSC-mediated suppression of Th17 differentiation from human naïve, cord blood-derived CD4^+^ T cells as well as IL-17A production by Th17 clones [20]. Going further, English et al. suggested that MSC modulate T cell polarization through a sequential process of regulatory T cell induction involving direct MSC contact with T cells followed by PGE2 and TGF-β1 expression [13]. The authors showed that PGE2 and TGF-β1 have non-redundant role in the induction of regulatory T cells. In the present study, we showed an increased expression level of TGF-β1 mRNA in MSC when cultured with either CD4^+^ T cells under Th1- or Th17-skewing conditions or with mature Th1 and Th17 cells, although to a lesser extend under Th1 conditions. Moreover, while TGF-β1 was up-regulated in MSC primed with Th17 conditioned media no change was observed with Th1 cell media. However, this increased expression level mediated by soluble factors contained in the Th17 conditioned media, was significantly lower compared with the TGF-β1 up-regulation in primed MSC upon a direct contact with the different subsets of T cells. Therefore, TGF-β1 could be rather expressed by MSC in response to a direct contact with T cells and thus participated to the education of immunosuppressive MSC. All together these results suggest that TGF-β1 expression could not explain the more potent inhibitory effect of MSC on Th1 cells.

Substantial evidence has revealed that PD-L1 can deliver an inhibitory signal to PD-1 expressing T cells, leading to suppression of the immune response by inducing apoptosis, anergy, unresponsiveness and functional exhaustion of T cells [41], [42]. Herein, we found that the increased expression level of PD-1 on T cells during the Th1 and Th17 differentiation processes was not affected by the presence of MSC. Moreover, although English and al. reported that PD-1 pathway was not essential for MSC mediated immunosuppression, more recently it has been shown that IFN-γ acts directly on MSC leading to an up-regulation of PD-L1 and, PD-L1 knockdown abolished MSC suppressive properties [11], [34]. These conflicting data on the role of PD-L1 on MSC immunomodulatory function might be related, in part, to the fact that in these two studies the authors used splenocytes or total lymphocytes. In our study, we observed an increased expression level of PD-L1 on MSC when cultured with 1) T cells induced to differentiate into Th1 and Th17 cells or the corresponding mature cells, 2) conditioned media obtained from cultures with mature Th1 or Th17 and, 3) upon treatment with IL-17, TNF-α and IFN-γ, alone or in combination. While there was no significant difference between treatments with IFN-γ alone or in combination with TNF-α and IL-17 in the percentage of MSC expressing PD-L1, we observed significantly higher PD-L1 MFI values when MSC were treated with the combination of cytokines. Our data suggest a synergistic effect of combining Th17 cytokines in increasing PD-L1 density on MSC compared with IFN-γ treatment. We also investigated the effect of other cytokines described to contribute to PD-L1 regulation and to be involved in the immunosuppressive function of MSC including IL-10 and IL-6 [43]–[46]. Although, a significant increase of IL-10 secretion was quantified in the co-culture supernatants of MSC with T cells induced to differentiate into Th1 or Th17, there was no change of PD-L1 expression on MSC after a treatment with IL-10 (data not shown). In parallel, we observed that IL-6 secretion was significantly increased in the supernatants from co-cultured MSC with CD4-Th1 or mature Th1 compared to MSC or Th1 cells alone. Upon treatment with IL-6, the percentage of MSC expressing PD-L1 was significantly increased (data not shown). Going further we demonstrated, that blocking PD-L1 expressed on MSC can restore the proliferation of differentiated Th17 cells. We also found that blockade of PD-L1 pathway suppresses the capacity of MSC to modulate the cytokine profile of fully differentiated Th17 cells by inhibiting the expression of the Th17 specific transcription factor RORγT and the production of Th17 specific cytokine IL-17. Therefore, we demonstrated that while PD-1/PD-L1 pathway plays a pivotal role in MSC-mediated suppression of Th17 cell proliferation and IL-17 production, it did not affect MSC suppressive effect on T cells induced to differentiate into Th1 and Th17 as well as on mature Th1 cells. The absence of a significant role of PD-L1 blocking antibody in the co-cultures of MSC with CD4^+^ T cells induced to differentiate into Th17 could be explained by the fact that the expression level of its receptor, PD-1, is increased gradually during the Th17 differentiation process to reach the maximum at day 4. Moreover, anti-PD-L1 neutralizing antibody did not modify MSC immunosuppressive properties on Th1 cells reinforcing the hypothesis that MSC exert their suppressive effects on Th1 cells through the secretion of soluble factor(s).

Taken together, our results demonstrate for the first time that mature Th17 cells produce cytokines that play a critical role in triggering the immunosuppressive effect of MSC through the up-regulation of PD-L1. They support a cell-to-cell contact dependence of MSC-mediated suppression on mature Th17 cells. The co-inhibitory molecule PD-L1 highly expressed by primed MSC is a key factor for their repressive activity on mature Th17 genetic program. The knowledge gained from such a study will facilitate our investigations using human MSC.
